# Systemic criticality—a new assessment concept improving the evidence basis for CI protection

**DOI:** 10.1007/s10584-021-03019-x

**Published:** 2021-03-01

**Authors:** Philip M. Kruse, Hanna C. Schmitt, Stefan Greiving

**Affiliations:** School of Spatial Planning, Institute of Spatial Planning (IRPUD), TU Dortmund University, Dortmund, Germany

**Keywords:** Critical infrastructure, Systemic criticality, Hydro-meteorological hazard, Flood protection, Risk assessment, System-of-systems

## Abstract

With high certainty, extreme weather events will intensify in their impact within the next 10 years due to climate change-induced increases in hazard probability of occurrence and simultaneous increases in socio-economic vulnerability. Data from previous mega-disasters show that losses from disruptions of critical services surpass the value of direct damages in the exposed areas because critical infrastructures [CI] are increasingly (inter-) dependent. Local events may have global impacts. Systemic criticality, which describes the relevance of a critical infrastructure due to its positioning within the system, needs to be addressed to reduce the likelihood of cascading effects. This paper presents novel approaches to operationalise and assess systemic criticality. Firstly, the paper introduces systemic cascade potential as a measurement of systemic criticality. It takes the relevance of a sector and the relevance of its interdependencies into account to generate a relative value of systemic importance for a CI sector. Secondly, an exemplary sectoral assessment of the road network allows reflecting the spatial manifestation of the first level of cascading effects. It analyses the impact of traffic interruptions on the accessibility of critical facilities to point out the systemically most critical segments of the municipal road network. To further operationalise the spatial dimension of criticality, a normative assertion determining the worthiness of protection of system components is required. A nationwide spatial flood protection plan incorporates this aspect in Germany for the first time. Its formal approval process was initiated in February 2020.

## Introduction

Convective storms are an intensifying hydro-meteorological hazard, which usually occurs in the second half of warm and hot days and result in thunderstorms and heavy precipitation (Deutscher Wetterdienst 2019; Klose 2008). Climate change is expected to exacerbate these hazards. Projections suggest that the intensity and frequency of extreme rainfall are to increase across most mid-latitude regions (Intergovernmental Panel on Climate Change [IPCC] 2014, 2018). Additionally, climate variability associated with the El Niño Southern Oscillation is projected to intensify as the temperature rises affecting global precipitation patterns (IPCC 2014; Klose 2008). An increasing number of hydro-meteorological extreme events can be observed globally since the 1970s (Munich RE 2019a; IPCC 2014) with losses being on an upwards trend. The five costliest convective storm events based on normalised overall losses have all occurred in the past decade (Munich RE 2019a).

Hydro-meteorological hazards can lead to far-reaching consequences for CI. For example, floods led to long-lasting disruptions on the high-speed rail service between Hanover and Berlin due to damages of a network segment after a dyke breach near Tangermünde in Saxony-Anhalt when the river Elbe exceeded its boundaries in June 2013. The track was closed for 5 months until November. As a result, 10,000 passenger and 3000 freight trains were rerouted, travel time increased by up to 180 min, and approximately a third of all passengers chose a different mode of transport. The upped travel time resulted in considerably higher energy consumption and costs and, consequently, economic losses (Deutsche Bahn 2014).

Data from recent mega-disasters prove the assumption that cascading effects, which cause disruptions in infrastructure networks and supply chains, result in more economic losses than direct damages within the exposed areas. For example, the floods in Bangkok in 2011 resulted in direct damages of 45.7 billion USD, yet the indirect economic losses due to supply chain disruptions associated with the event are at approximately 259 billion USD (AON Benfield 2012; Cookson and Davies 2011). Hurricane Sandy caused direct damages of about 50 billion USD in New York in 2012. Again, supply chain disruptions caused indirect economic losses of circa 250 billion USD (AON Benfield 2013). Also, the current Covid-19 pandemic is costing between 8.1 and 15.8 trillion USD globally (World Economic Forum 2020), primarily because of disrupted global supply chains (Statista 2020).

These examples show the urgency of addressing cascading effects in the context of climate adaptation and disaster management. Climate change itself and related weather extremes are additional triggers for cascading effects that inflict the functionality of CI systems. Considering cascading effects is necessary for adaptation to be successful and to reduce potential impacts and related losses. The reason for these indirect losses is the increasing complexity and interdependence of critical infrastructures. By definition, CI are organisations and institutions of importance to the state community because their impairment could lead to prolonged supply bottlenecks and inflict public safety. They are constituted by basic technical infrastructures and socio-economic service infrastructures. Criticality measures the importance of an infrastructure based on the consequences of its impairment (BMI 2009).

This paper covers systemic criticality, which addresses the effects of infrastructure failure on supply dependencies within the CI system. For example, if a facility is damaged by a natural hazard, this will trigger cascading effects to other facilities, networks, and even sectors. To reduce the economic losses caused by disasters in the face of climate change as targeted by Sustainable Development Goal [SDG] 11 (United Nations 2015), a better understanding and operationalisation of systemic criticality appears to be necessary. The first priority of the Sendai Framework for Disaster Risk Reduction (United Nations Office for Disaster Risk Reduction [UNDRR] 2015) emphasises this need further. There is still dissent as to how criticality is to be understood and, thus, operationalised (Katina and Hester 2013). Existing approaches are often not practical, and further research and development are required to implement criticality assessments in decision-making processes (Fekete 2011, 2018; van Eeten et al. 2011). The operationalisation of systemic criticality is essential for management approaches, as these address the worthiness of an infrastructure’s protection, which is derived from its systemic relevance. Therefore, this paper asks:

- How can systemic criticality be operationalised?
- How can the results of criticality assessments be used to improve CI protection?

This paper contributes to the ongoing discussion about the conceptualisation of criticality by laying the theoretical groundwork in Section 2. Section 3 introduces the operationalisation approach of systemic cascade potential to measure systemic criticality and applies it to a spatial context by performing a sectoral criticality analysis of the road network. A context-specific management approach is subsequently discussed in Section 4. Sections 5 and 6 discuss and conclude this paper.

## Criticality: an inherent feature of CI

CI protection and maintenance of service are of such significance that one of the global goals of the Sendai Framework for Disaster Risk Reduction is to “[s]ubstantially reduce disaster damage to critical infrastructure and disruption of basic services […] by 2030” (UNDRR 2015). Accordingly, the protection of CI is also subsumed under SDG 11. One of its targets is to “increase the number of cities […] adopting and implementing integrated policies and plans towards […] mitigation and adaptation to climate change, resilience to climate change, and to develop and implement, in line with the Sendai Framework for Disaster Risk Reduction 2015 - 2030, holistic disaster risk management at all levels” (UNDRR 2015) by 2020. The protection of infrastructures and the maintenance of critical services is also relevant to another target of SDG 11 aiming at “substantially [decreasing]” (United Nations 2015) the economic impacts from hazardous events by 2030.

The European Council understands CI as an essential precondition for the functioning of societies. According to Council Directive 2008/114/EC, CI are “an asset, system or part thereof […] which is essential for the maintenance of vital societal functions, health, safety, security, economic or social well-being of people, and the disruption or destruction of which would have a significant impact […] as a result of the failure to maintain those functions”. The infrastructures of European significance are constituted by the energy and transport sectors (Council Directive 2008/114/EC).

To understand the complexity of the CI system and the internal and external effects of service disruptions, it is important to understand criticality, which the German Federal Ministry of the Interior, Building, and Community [BMI] defines as “a relative measure of the importance of an infrastructure in terms of the consequences of a disruption or failure for the security of supply of essential goods and services to society” (BMI 2009). It is a characteristic innate to CI, which the legislator defines as “organisations and institutions that are important for the state community and whose failure or impairment would result in sustained supply bottlenecks, major disruptions to public safety or other dramatic consequences” (BMI 2009). According to §§ 2–8 of the Ordinance on the Identification of Critical Infrastructures in accordance with the Act on the Federal Office for Information Security (BSI-Kritisverordnung) [BSI-KritisV], the German Federal Government not only defines the energy and transport sectors as critical but also the water, nutrition, information technology and telecommunications, and health, as well as the finance and insurance sectors. The German National Strategy for the Protection of Critical Infrastructures [CI Strategy] further extends these sectors by including the emergency services, administrative institutions, the media, and cultural assets (BMI 2009).

The CI strategy’s definition implies several characteristics of criticality. For instance, it is “a relative measure” (BMI 2009). Criticality is not an absolute value but must be considered relative to its context. This context may be determined by, among others, legal obligations, explored administrative levels, the boundaries of the investigated area, or the considered infrastructure sectors and their worthiness of protection (Engels 2018; Folkers 2018; Bouchon 2006). Only in this context, propositions on the criticality of a system component, may it be a facility, network segment, or subsector, can be concluded (Engels 2018; Lukitsch et al. 2018). For example, a segment of a motorway is more critical than a segment of a high street relative to all streets within given boundaries, because a disruption would have more extensive implications for a larger number of people and businesses. However, when only considering the municipal streets within the same boundaries, the high street segment’s criticality may be higher because the investigated context is different (BBK 2017; Birkmann et al. 2016; Bouchon 2006).

What is the effect when an infrastructure component breaks down, or services are disrupted? Lukitsch et al. (2018) argue that this question, and thus the question of how to assess criticality, can be answered from a deficiency-oriented perspective. Through this lens, criticality is assessed by investigating the impact of a defective component on the system and population. This approach shows that infrastructures are a considerable contributing factor impacting a society’s susceptibility to natural hazards. However, identifying the weak links of the infrastructure system can also be an opportunity to invest strategically in reducing a community’s vulnerability (Lukitsch et al. 2018).

The emphasis on consequences implies an understanding of criticality that Lukitsch et al. (2018) coin as function-oriented. The core assumption of this use of the term is that “function [...] is the result of an interplay of individual components” (Lukitsch et al. 2018) within the infrastructure system. In this context, function or functionality is defined relative to a given or expected supply of services or goods. For example, a functional electricity supply network provides energy for all households connected to it and is expected to perform without disruption. The opposite of a functioning is a malfunctioning system, i.e. when infrastructures break down, and services are disrupted. The criticality of an infrastructure system component can be assessed by the consequences of its failure. The previously mentioned definition of critical infrastructure alludes to these consequences when it mentions that a disruption of infrastructure services may lead to “sustained supply bottlenecks, [and] major disruptions to public safety” (BMI 2009). These consequences may either have direct or indirect implications for society as well as other CI systems. To stick with the example of electricity supply, a transducer breaking down would have a direct impact on the households, businesses, and public buildings connected to this facility. Considering a facility-based perspective in this case, criticality is comparatively higher the more households, enterprises, and public buildings are connected to a specific transducer and would, therefore, be affected by a blackout if it broke down. System-internal implications of supply disruptions can be accounted for when addressing criticality from a systemic perspective. For example, when a component of the electricity supply system breaks down, this may lead to a partial or complete blackout heavily impairing any other electricity-related CI system such as ICT. Besides these inter-sectoral cascading effects, the impairment may have an additional intra-sectoral character; i.e. the blackout inflicts the electricity sector itself by debilitating the black start capacity of power plants.

While climate change indeed increases disaster risk, it does not directly implicate criticality itself. Thus, the concepts of risk and systemic criticality must be brought together. Systemic criticality cannot be added as an additional factor of the risk formula nor can it be added as another component of vulnerability, because disaster risk and vulnerability are place-based concepts (Greiving et al. 2016), while systemic criticality is not. Both, risk and systemic criticality, are to be analysed separately to guarantee consistent results without mixing up the functional character of systemic criticality with vulnerability. The result of the systemic criticality analysis can be integrated during the risk evaluation to consider the system-internal implications of hazards adequately. This addition is imperative to formulate effective management strategies and policies to help society cope with climate change-driven risks.

## Operationalising criticality

To be able to address and adequately manage criticality, it needs to be assessed and evaluated. State-of-the-art is to integrate criticality into risk evaluation through the approximation of a risk aversion factor, e.g. as done by Swiss authorities. The aversion factor is a multiplier, which estimates the economic loss caused by cascading effects by multiplying direct damages with a logarithmic factor: the higher the damage potential of the event, the greater the multiplier (Bundesamt für Bevölkerungsschutz [BABS] 2008). However, this approach is solely based on the damage potential of the event and disregards the specific characteristics of extreme events which influence cascading effects. Therefore, this section discusses a novel approach to operationalise systemic criticality so it may contribute to a more case-specific risk evaluation. It also points out how the spatial manifestation of a, thus far, non-spatial concept may be addressed.

### Conceptual operationalisation of systemic criticality

The German CI strategy states that “an infrastructure possesses a systemic criticality if it is of particularly high interdependent relevance due to its structural, functional, and technical positioning in the overall system of infrastructures.” (BMI 2009) Thus, systemic criticality reflects a system-internal perspective on CI as functional systems and their dependence on one another. The relevance of analysing systemic criticality increases as extreme events and other external hazards also affect the internal system of CI, potentially triggering cascading effects, which should be anticipated through adequate policies.

#### Methodology: operationalising systemic criticality

The operationalisation of systemic criticality faces several challenges. First, CI systems have an uncountable number of (inter-)dependencies. There are external dependencies of society on CI services, but more importantly, also internal (inter-)dependencies of CI systems relying on the services of other systems. For example, the water supply systems rely on electricity for the operation of pumps. The (inter-)dependencies and cross-sector services result in a tightly woven network of CI systems (Katina and Keating 2015; Pescaroli and Alexander 2015; Uday and Marais 2015) making them more than the sum of their parts (Vester 2015; Eusgeld et al. 2009).

Second, CI are bipolar as they are both physical and functional (service-providing) structures (Zimmerman et al. 2017; Katina and Keating 2015; Bouchon 2006). The functional characteristics of CI are hardly tangible as they are neither place-based nor do they operate within administrative boundaries (and legislative competencies of single stakeholders). However, their physical characteristics pose a challenge as well, because there is an unmanageable number of physical facilities and their components (Pinto et al. 2012).

Third, the CI system is dynamic. It continually evolves due to technological improvements and new dependencies making it unpredictable and never fully known (Zio 2016), as some characteristics of the system may only become apparent during an impairment or CI failure (Hellström 2007). These characteristics make CI a complex system-of-systems [sos] (Katina and Hester 2013; Eusgeld et al. 2011).

CI being a complex sos leads to challenges in the operationalisation, because established concepts like risk and vulnerability cannot address the systemic criticality of CI systems and subsystems (Katina et al. 2014; Hellström 2007; Bouchon 2006). There are again three reasons for this statement: First, classical risk approaches are probabilistic and rely on linear cause-effect relationships that do not exist in complex systems (International Risk Governance Council [IRGC] 2018; Libbe et al. 2018; Pinto et al. 2012, referring to Pinto et al. 2012). Therefore, no assertions about the probability of occurrence, extent of damage, and uncertainties (and hazards) can be made regarding system failures (Fekete 2011; Di Mauro et al. 2010; Hellström 2007). Furthermore, such parameters cannot illustrate what criticality actually means, namely positive and negative, relative relevance (Lukitsch et al. 2018; Engels 2018; Engels and Nordmann 2018). Second, risk and vulnerability are place-based concepts, but the CI sos is—at its core—of functional character (IRGC 2018). Third, (inter-)dependencies require “an assessment of […] the entire network of an infrastructure (e.g., electricity) and its potential cascading effects on other infrastructure systems” (Birkmann et al. 2016). Even if risk analyses were carried out for single facilities and their parts through systemic computational modelling, the system state and embeddedness in the greater sos could not be realised for more than a few directed dependencies, even with computational support (Eusgeld et al. 2009; Haimes 2009).

The complexity of the CI sos and the lack of approaches accounting for the systemic complexity of (inter-)dependencies (Katina et al. 2014) most often result in chronic underestimation of potential cascading effects (IRGC 2018; Eusgeld et al. 2011), which in turn results in incomplete and potentially misleading policies (Garschagen and Sandholz 2018; Bouchon 2006). Therefore, an approach capturing (inter-)dependencies between the subsystems of the sos is required to make systemic criticality measurable.

##### Approach of “systemic cascade potential”

The approach for operationalising systemic criticality presented in this paper stems from an ongoing PhD (Schmitt, H.C.) in spatial planning. The “systemic cascade potential” approach expresses the possibility and strength of a cascading effect potentially propagating through the CI sos over time. It encompasses the possibility and strength of cascading effects from a solely systemic, i.e. functional, system-internal, and non-spatial, perspective. It aims at uncovering the sos and its potential cascading effects under certain aspirations. First, the operationalisation approach shall be able to encompass the whole CI sos, its subsystems, and their connectivity as comprehensively as possible. Second, the sos shall be reduced in complexity so that it may be (resource-efficiently) transferred to different spatial levels and contexts. Thus, the approach needs to be generic.

Based on these aspirations, conventional interdependency analyses (e.g. Laugé et al. 2015; BABS 2010) are extended into two factors and four parameters, encompassing the individual CI subsystems and their interconnectivity. Factor 1 reflects the “relevance of subsystems” and is parameterised by the (inter-)dependencies of each subsystem to all others. The first parameter of factor 1, the “degree of subsector connectivity”, reflects on the number and the character (unidirectional or bidirectional) of dependencies. The second parameter “centrality” includes the type of dependency (direct or indirect) and the closeness centrality every subsystem has, based on its average path distance. Factor 2 focuses on the “relevance of dependencies” and is parametrised by the intensity of a potential cascading effect over time. Therefore, the third parameter measures the “intensity of a potential cascade” by approximation of the severity of potential impairments. The last parameter is the “propagation speed”, which reflects on the severity of potential impairments over time.

**Fig. 1:**
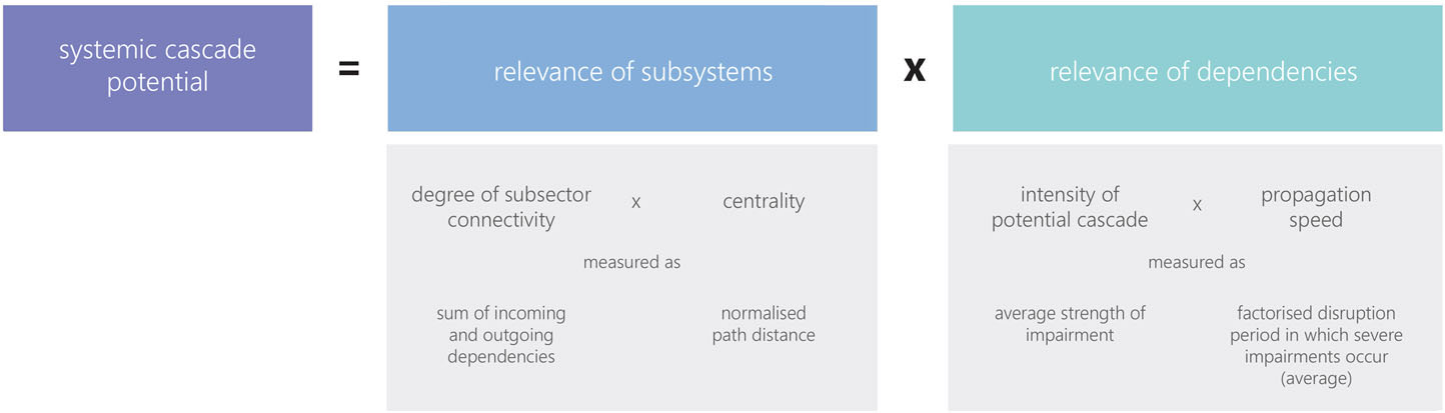
Factors to operationalise the systemic cascade potential (own depiction based on Schmitt 2019)

As Fig. 1 displays: Factor 1 is the product of the degree of subsector connectivity (sum of incoming and outgoing dependencies) and the centrality of the subsector in the sos (averaged, normalised path length, calculated in a network visualisation programme). Factor 2 is the product of the intensity of a potential cascade (average impact of outgoing dependencies) and the propagation speed (weighted disruption period causing severe impairments on average).

##### Application to a German national context

In order to apply this generic approach, which is scalable to any spatial level and context, some assumptions must be made. First, the investigated “subsystems” need to be defined. For an application on the German context, the 29 subsectors of the CI Strategy were used. Second, disruption periods need to be selected. These may vary with different research interests, and they were set to 4 h, 24 h, 4 days, 2 weeks, and 6 weeks within this context to reflect a broad spectrum of responsibilities and stakeholders. Last, the data collection method needs to be selected. In this case, online surveys collected responses from expert interviews.

Experts to be interviewed were selected following specific targeting criteria. First, the experts’ organisation had to represent an objective federal perspective and ideally possess competences related to CI. Consequently, most experts represented federal authorities or nationwide associations. Second, the targeted experts have expertise and experience in CI management. Based on these targeting criteria, experts were (deliberately) contacted via email with a personalised link to the survey of “their” subsector in the online tool SoSci-Survey. Due to reasons of anonymity and representativeness, there had to be at least three responses from each of the 29 subsectors (*n*_min_ = 87).

The survey was based on a fictional setting, assuming the impact of a sudden, total, nationwide failure of each and every subsector. The interviewee was exclusively asked about their particular portfolio. It encompassed three questions. The first two multiple-choice questions asked for the number of outgoing and incoming dependencies to gather the information required for factor 1. The third question investigated the intensity of (the previously selected) incoming dependencies for the five disruption periods through a visual analogue scale in order to gather the information for factor 2. Additionally, qualitative information was surveyed to interpret the results.

The survey took place within 8 days in spring 2019 with more than 100 experts participating. The obtained data allows for several analyses, e.g. for issuing subsector profiles contributing to a deeper understanding of single subsectors, but also the sos as a whole (e.g. through network and cascade diagrams). As this article focuses on the operationalisation of systemic criticality, Table 1 shows an excerpt from the calculation of the systemic cascade potential.

**Table 1:** Excerpt from the calculation of the systemic cascade potential in German CI subsectors (based on Schmitt 2019)

Subsector	Connectivity	Closeness centrality	Factor 1:	Intensity of potential cascade	Propagation speed	Factor 2:	Systemic cascade potential
			Subsector relevance			Relevance of dependencies	
Calculation	Sum of incoming and outgoing dependencies	Normalised path distance	Connectivityxcloseness centrality	Ø strength of impair-ment	Disruption period causing Ø severe impairment	Ø severity xdisruption period	Factor 1xFactor 2
Electricity	102	0,97	98,94	3,84	× 16	61,38	6.073
IT	83	0,97	80,51	3,76	× 16	60,13	4.841
Telecommunication	82	0,97	79,54	3,63	× 16	58,14	4.624
Civil protection and emergency services	66	0,67	44,22	3,38	× 16	54,02	2.389
Water supply	52	0,78	40,56	3,56	× 16	56,96	2.310
[…]	[…]	[…]	[…]	[…]	[…]	[…]	[…]
Insurance	46	0,57	26,22	2,39	× 2	4,78	125
Cultural Property	33	0,25	8,25	2,6	× 4	10,4	86
Domestic Shipping	26	0,6	15,6	2,36	× 2	4,73	74

#### Results: measuring systemic criticality

A calculation of the systemic cascade potential becomes possible due to aggregation and averaging the survey data (parameters 1, 3, 4) and calculating closeness centrality through network analysis (parameter 2). Table 1 presents an excerpt of the calculation for the German CI subsectors.

The results show a great range between subsectors with the highest and the lowest systemic cascade potential, leaving electricity with about 80 times the systemic cascade potential compared to domestic shipping. These results may further be categorised, similar to the Swiss CI strategy (s. BABS 2010), prioritising systemic criticality and transferring it to suggestions for the worthiness of protection. The exact demarcation of categories is a normative decision.

### Spatial operationalisation of systemic criticality

While criticality is not place-based, system-internal cascades still manifest themselves spatially. The question remains as to how the functional character of systemic criticality can be operationalised to be represented and translated into spatial models. The following section attempts to do just that by assessing the cascades following traffic interruptions following breaking branches and trees during extreme storms.

#### Methodology: sectoral assessment of the road network’s criticality

This method was developed during the BaumAdapt project, which is funded by the German Ministry for the Environment, Nature Conservation, and Nuclear Safety within the framework of the German Strategy for Adaptation to Climate Change. The project was conceptualised in the wake of storm Ela in June 2014, which ranks among the five most severe convective extreme events in Germany since 1980 (Munich RE 2019b). Traffic infrastructure was severely affected by the direct impact of the storm resulting in prolonged interruptions of road and rail traffic (Haering and Bösken 2014). The systemic criticality of road network segments can be considered to prioritise preventative actions, such as adapting the urban tree stock to evolving climatic conditions. As there was no approach to such an assessment, a new method was developed.

**Fig. 2:**
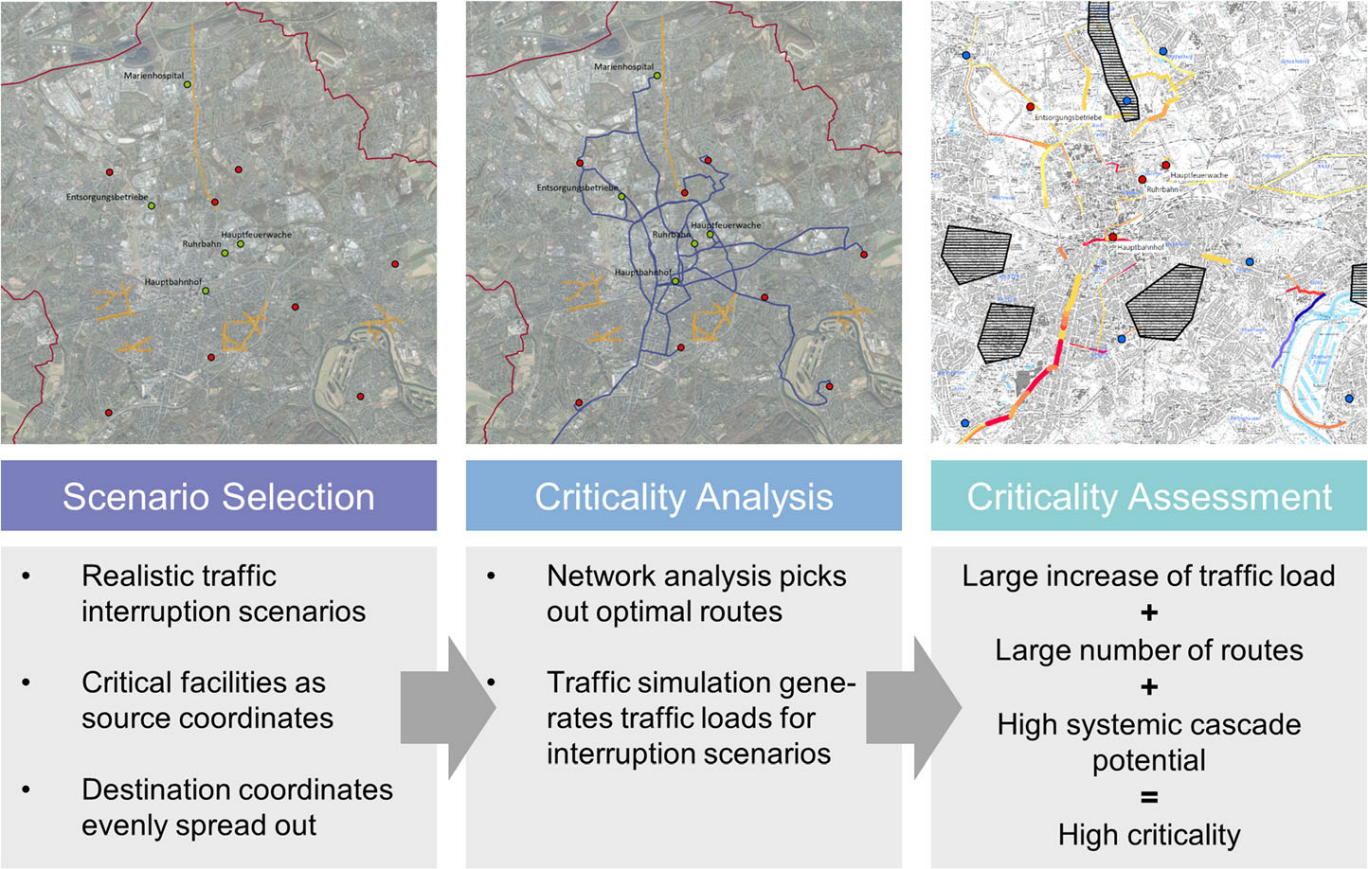
Multi-step approach for a sectoral criticality analysis of road traffic (own depiction)

It analyses the systemic criticality of the municipal road network segments by evaluating the effects of selected traffic interruptions on the accessibility of critical facilities. The method applies a multi-step approach (s. Fig. 2) to assess the change of traffic load on routes between two coordinates in a city for various interruption scenarios. The coordinates of these origin-destination-relationships each consist of a critical facility and a residential building block. The method assumes that a network segment is the more critical, the more the traffic load increases and the greater the number of origin-destination-relationships it is relevant for. It utilises the traffic assignment problem: Travel time, and thus traffic load, is linked to individual route choices. These decisions depend on network congestion, which is a function of the choices of all other network users (Ukkusuri and Yushimito 2009). Traffic models attempt to solve this problem.

First, relevant origin-destination-relationships and realistic traffic interruption scenarios need to be selected. In the BaumAdapt project, this selection was made in a consultative process with municipal actors involved in disaster management and the maintenance of the urban tree population. Given the project context, the origin coordinates reflect facilities required for the response to the impact of a storm on urban trees and forests, which the interruption scenarios also reflect. These facilities represent other CI sectors, which could be adversely affected by service disruptions and delays within the transport network. The second coordinate of the origin-destination-relationships needs to be spread across the assessed area to analyse the accessibility of each critical facility from throughout the city. In the case of BaumAdapt, the most populous building block of each city district was used; thus, guaranteeing coordinates are spread out evenly. With five critical facilities selected and nine residential building blocks, 45 origin-destination-relationships were analysed in six scenarios during the project.

Second, a criticality analysis is run applying GIS-based network analysis and a traffic model. The optimal route between each building block and each critical facility is calculated for each scenario using the ArcGIS Pro Network Analyst (Esri 2020). These routes are used to identify the relevant network segments from the traffic model. A traffic model breaks the road network down into nodes, i.e. road junctions, and sections, i.e. the road segments between two junctions. Traffic is fed into this network through cells. Each cell contains statistical data relevant to traffic generation, e.g. number of residents or employees. Traffic gravitates between these cells based on the embedded data and generates traffic loads for each section (Helmert and Henninger 2017). A traffic simulation is run to generate traffic loads for the 0 scenario and all interruption scenarios. The 0 scenario describes a network without any road closures, thus free-flowing traffic without interruptions and delays. The applied traffic model only included the municipal road network. While external traffic flows are accounted for by cells, there are no nodes or sections outside the municipal borders. Based on the results of the network analysis and traffic simulation, all relevant traffic loads can be exported into a spreadsheet and compared to the loads of an uninterrupted network. This difference is one of the indicators applied to assess the criticality of road segments.

The final step concerns the criticality assessment. Based on the spreadsheet, the road segments most relevant in each scenario can be identified. This relevance originates from the increase of traffic load and the number of routes running over this network segment. Criticality increases, the higher the traffic load and the greater the number of routes. All scenario evaluations are compared as the validity of the assessment improves with the number of scenarios reflecting similar assertions. The validity further increases with a greater number of origin-destination-relationships and interruption scenarios. Additionally, the systemic cascade potential (s. 3.1) of each critical facility, which represents a CI subsector, should be considered in the criticality assessment to address additional system-internal cascading effects.

#### Results: the systemic importance of road network segments

The sectoral criticality assessment of the road network allows making assertions towards the systemic importance of a segment that is relevant for the accessibility of critical facilities. These are the network segments with an especially large increase in traffic load and that are part of multiple origin-destination-relationships. The validity of these assertions can be increased, the higher the number of simulated scenarios and the higher the number of origin-destination-relationships confirming them.

The analysis results show three network segments that have a comparatively higher systemic criticality than the rest of the network for three different reasons:

1. The primary connection between the south-eastern boroughs and the city centre is blocked, leading to an increased traffic load on the diversion route. At up to 15,000 more vehicles per hour, this is the highest increase on any relevant network segment.
2. The connection between the south-western boroughs and the city centre only shows a traffic load increase of up to 7000 vehicles per hour, which ranks among the medium values on the scale. However, it is a relevant network segment for twelve origin-destination-relationships, which is more than can be observed with any other segment.
3. The third segment is only relevant to connect to one critical facility and shows a medium-high traffic load increase of up to 6000 vehicles per hour. The reason for its high systemic criticality is the importance of the facility, which is accessed from the road, i.e. the municipal waste management company. Most heavy machinery to respond to the storm impact on the urban environment is stored there; thus, inaccessibility could start cascading effects affecting other sectors.

Which of the three indicators - traffic load increase, number of relevant origin-destination relationships, or the systemic cascade potential - ranks higher cannot be determined on the factual level and requires a normative decision.

Still, the sectoral analysis is limited and cannot operationalise the real complexity of the CI sos and its systemic criticality. It only considers the road network; thus, assertions towards systemic relevance can only be made regarding one subsector. The limitation is further determined by the boundaries of the spatial scope, as only the road network within the municipal boundaries is considered. However, the spatial scope can be scaled by applying a traffic model on a different scale. Albeit its limitations, this assessment indeed allows operationalising systemic criticality in its spatial context. Previous assessments of the transport network neglect the systemic component or only address facilities, such as tunnels or bridges, without considering the systemic criticality of the network itself (e.g. Ukkusuri and Yushimito 2009, Friedman et al. 2006). The method is further limited as it only addresses the first level of cascading effects from a storm-related interruption of the network. Further system-internal consequences cannot be operationalised through this approach and require consideration of the systemic cascade potential of sources and destinations. The result is evidence that can feed into decision-making processes and support the prioritisation of measures to prevent infrastructure service disruptions and making society more resilient to extreme weather along the way.

While criticality may be accounted for in analyses on the local or regional level, the question remains whether a result is eventually valid. CI networks and dependencies between subsectors do not end with administrative boundaries. Instead, they are deeply interconnected with the critical supply networks of national and supranational concern. This issue does not only concern the assessment of criticality but also how it is managed in the context of hydro-meteorological hazards as the following section explains.

## Discussion of legal implications and changes in risk management

Any spatial risk management needs to be place-based. Spatial planning authorities are only legally responsible to manage the land use of their area of responsibility. They can and should take the physical component of CI, thus, the consequence-based element of criticality into account. However, cascading effects may take place outside the defined planning area, while spatial planning just operates for a specific territory, which often does not capture entire networks. Moreover, planning authorities do not have appropriate information on systemic criticality at hand. Consequently, the systemic focus of criticality runs counter to the areal-oriented view of spatial planning (Greiving et al. 2016).

This dilemma does not mean that systemic critically cannot be addressed by spatial planning. Systemic criticality should be addressed for infrastructure-related decisions considering the entire network on large-scale territorial levels, such as the European Union (e.g. Trans-European Networks [TEN]) or national levels (e.g. Federal Transport Infrastructure Plan; Bundesverkehrswegeplan [BVWP]).

**Fig. 3:**
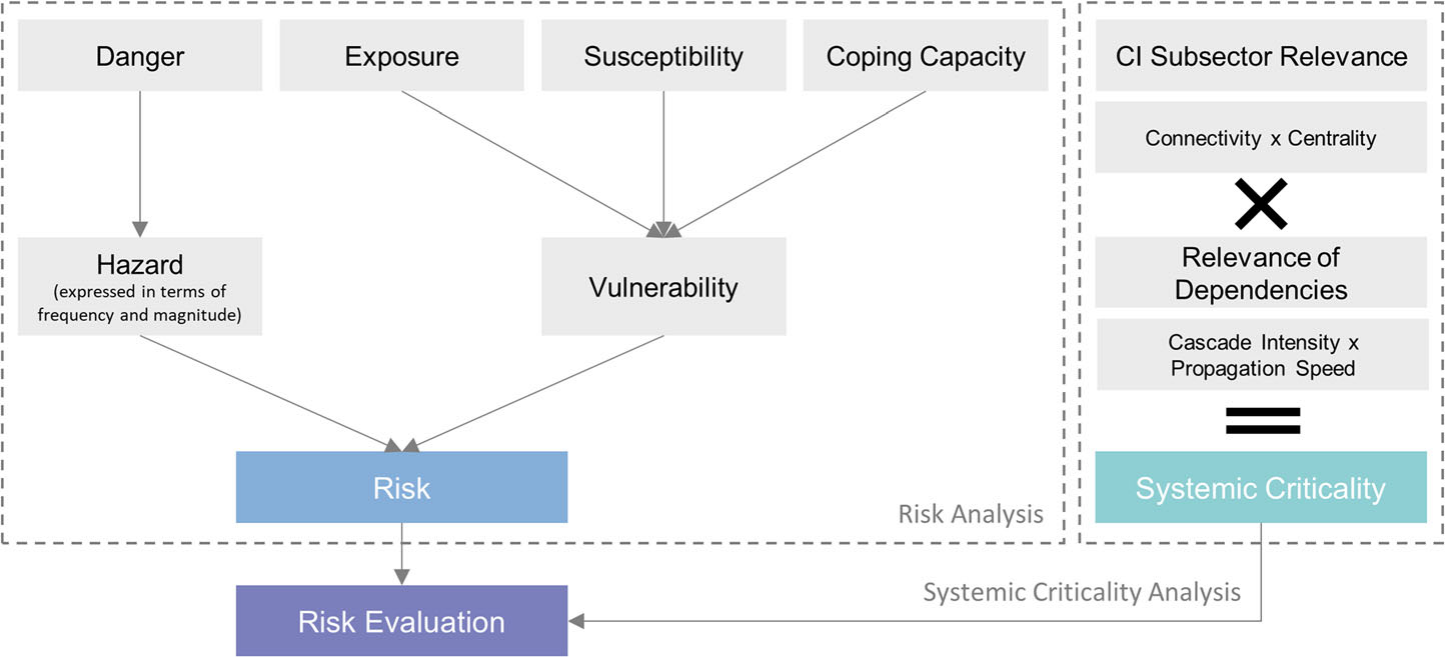
Concept of combining risk and criticality analyses in risk evaluation (own depiction)

Figure 3 outlines that the conventional concept of risk assessments should be complemented by a systemic perspective beyond the place-based understanding of vulnerability. While risk assessments focus on direct damages, systemic criticality analysis needs to consider the entire CI sos and its interconnections. Both assessment results should then be jointly evaluated to manage potential impacts of hazardous events by determining the worthiness of protection of settlements and infrastructures depending on their criticality.

The legal implications of the criticality concept presented by this paper show that this concept is not just of theoretical nature, but it is a real-world discussion of European relevance. A new planning instrument is worth mentioning as it follows this new understanding of criticality for the first time. With the amendment of § 17 (2) clause 1 of the Federal Regional Planning Act (Raumordnungsgesetz) [ROG] in 2017, the federal legislator has enabled the BMI to establish cross-federal state regional plans for flood protection in the form of a statutory ordinance. Flood protection addresses coastal, fluvial, and pluvial flooding in this context.

On this legal basis, a test plan and the conceptual design of a federal spatial plan for a nationwide spatial flood protection plan (Bundesraumordnungsplan Hochwasserschutz [BRPH]) have been developed. Opposed to the legislator’s original intention of catchment-specific plans, the upcoming plan covers all of Germany to address CI networks comprehensively, which are not limited by the borders of federal states and river basins.

This test plan consists of three chapters:

1. The plan establishes a risk-based approach providing a framework for the further subject matter of the plan. Currently, flood protection in Germany is based on hazard zones demarking the areas potentially inundated during a 1:100-year flood event. This approach does not include the hazard intensity (in terms of flood depth or velocity) and the vulnerability of exposed land uses. These aspects must be considered when demarking flood risk zones in the future.
2. Critical infrastructures and establishments that pose a particular risk: This chapter comprises the regulated matters based on the worthiness of protection, which has been lacking in spatial planning so far. Spatial planning must weigh the protection of an infrastructure higher against other concerns and interests (see also Riegel 2015) now. Moreover, establishments according to Art. 3 § 12 Directive 2012/18/EU (SEVESO III Directive) operating with dangerous substances present in one or more installations must be planned and constructed outside areas prone to flooding with a low probability.
3. Preventive flood protection: This chapter addresses the existing key priorities for action laid out by the Ministerial Committee on Regional Planning. In shaping them, the framework requirements of chapter 1 must be considered, particularly the risk-based approach. For example, facilities whose users depend on external support in case of flooding, e.g. hospitals, schools, and homes for the elderly, must be built outside areas prone to flooding with a low probability.

Regarding CI protection, the concept of worthiness of protection is based on existing European legal acts. Systemic criticality is normatively determined in this context: Infrastructure networks contributing to the attainment of major Union objectives, as set out in, among others, the Europe 2020 Strategy and the Commission White Paper “Roadmap to a Single European Transport Area – Towards a competitive and resource-efficient transport system” (“the White Paper”) require specific protection. Among these objectives are the smooth functioning of the internal market and the strengthening of economic, social, and territorial cohesion (European Commission 2011).

The primary European legal basis for critical infrastructures is the “Trans-European Networks” chapter in the Treaty on the Functioning of the European Union (2007/C306/01) [Treaty of Lisbon]. Article 170 § 1 states: “To help achieve the objectives referred to in Articles 26 and 174 and to enable citizens of the Union, economic operators and regional and local communities to derive full benefit from the setting-up of an area without internal frontiers, the Union shall contribute to the establishment and development of trans-European networks in the areas of transport, telecommunications and energy infrastructures.”

Council Directive 2008/114/EC on the protection of European critical infrastructures lays down the secondary legal basis. The directive is reasoned by the fact that “[t]here are a certain number of critical infrastructures in the Community, the disruption or destruction of which would have significant cross-border impacts. This may include transboundary cross-sector effects resulting from interdependencies between interconnected infrastructures.”

Consequently, Art. 2 b) defines European critical infrastructure [ECI] as follows: “‘ECI’ means critical infrastructure located in Member States the disruption or destruction of which would have a significant impact on at least two Member States. The significance of the impact shall be assessed in terms of cross-cutting criteria. This includes effects resulting from cross-sector dependencies on other types of infrastructure.” However, the directive just covers transport and energy infrastructures because of the given legal basis for these sectors as laid down by Art. 170 Treaty of Lisbon.

However, spatial and factual definiteness or at least determinability are a condition for setting-up legally binding spatial objectives in the context of the BRPH. The regulations (EU) No. 1315/2013a of the European Parliament and of the Council of 11 December 2013 on “Union guidelines for the development of a trans-European transport network” and (EU) No. 347/2013 of the European Parliament and of the Council of 17 April 2013 on “Guidelines for the trans-European Energy infrastructure” offer this spatial and factual determinability. They determine key elements of the trans-European transport network and “projects of common interest” of the European energy infrastructure whose protection matters most from a European perspective due to their relevance for the functioning of the single market. This importance can be applied as a proxy indicator for their extraordinary systemic criticality.

Regulation (EU) No. 1315/2013a determines the core elements of the Trans-European Transport Network visually and lists each network element in tables, which indicates the legally necessary spatial definiteness. It enables the German federal legislator to lay down spatial objectives aimed at the protection of these core elements against impacts of even rare flood events. Regulation (EU) No. 347/2013b establishes nine “priority corridors” of the European energy infrastructure and identifies projects of common interests within these corridors. Different to the transport infrastructure, the element of factual definiteness is of greater importance as each project of common interest is described in detail by annex III of the regulation.

Due to the given spatial and factual determinability, the test plan came up with two legally binding spatial objectives that point at the worthiness of protection of the core elements of the TEN-T (transport) and TEN-E (energy) networks:

**Table:** 

The transport infrastructure elements that are part of the core network of TEN-T laid down by Regulation (EU) 1315/2013a must be built outside areas prone to floods of a low return period. Exceptions can be allowed for infrastructure measures that must be built near water or cross water, provided that they are adapted to the respective flood hazard. Projects of common interest (PCI) laid down by Regulation (EU) 347/2013b must be built outside areas prone to floods of a low return period. Exceptions can be allowed for infrastructure measures that must be built near water or cross water, provided that they are adapted to the respective flood hazard or that the measures are not vulnerable to the effects of flooding.	

Moreover, the test plan contains spatial principles targeting already existing components of the transport and energy system. These principles are not legally binding but put specific weight on particular concerns like flood protection. Specific attention must be paid to flood protection when extending, renewing, or upgrading existing network elements.

A gaming simulation to test the application of the BRPH took place in September 2019 in two regions (coastal and inland) in cooperation with representatives of regional and local planning authorities. Gaming simulations have been widely used in urban planning and since been introduced to other topics, such as risk management. The objective of a gaming simulation is to gain insight into real-life problems and relations between actors, especially related to new challenges, new analysis methods, new management approaches, or even new laws (Meier and Duke 2007). The essential purpose of the gaming simulations was an ex-ante assessment of necessary amendments of existing regional and local plans against the proposed objectives of the BRPH. The gaming simulations have confirmed that the test plan meets the necessary legal conditions laid down in § 17 (2) clause 2 ROG. The foreseeable impacts on regional and local plans seem to be manageable. Infrastructure planners and operators were part of the gaming simulation and approved the appropriateness and applicability of the previously mentioned objectives and principles.

## Discussion

The first research question is answered with the findings in the third section of this paper. Systemic criticality can be expressed at a factual level by identifying the systemic cascade potential of infrastructure sectors. This approach (s. 3.1) reduces the complexity and intangibility of the CI sos. It contributes to understanding system-internal dependencies and may be used to anticipate scenarios on potential effects from internal and external hazards. This way, the approach also raises awareness of outgoing dependencies, contributes to stakeholder cooperation, and may support policymaking by classifying the systemic cascade potential into classes of the worthiness of protection.

However, the approach comes at the price of abstractness and without a spatial dimension, i.e. not readily applicable for spatial planning or civil protection. Therefore, the sectoral criticality analysis of the road network (s. 3.2) attempts to operationalise systemic criticality in a way that allows investigating how it manifests itself in a spatial context. It contributes to the understanding of how hydro-meteorological hazards put the CI system at risk and, thus, may put the entire society at risk through cascading effects. The results inform about the most critical segments of the road network to uphold other services within the CI sos. This evidence can feed into decision-making processes and support the prioritisation of measures preventing infrastructure service disruptions and making society more resilient to hydro-meteorological hazards.

The CI system is complex. Apart from its purpose of supplying society with critical services, it is characterised by dependencies and interdependencies within and between different sectors. A component failure can have far-reaching consequences for the sos and have an impact outside the area initially exposed to the hazard. However, the assessment simplifies the CI system to the degree that only considers the first level of cascading effects by analysing the accessibility of critical facilities during traffic disruptions. To make assertions on systemic effects following the disruption of the services of these facilities is not possible.

To answer the second research question, a normative judgement is required to further operationalise the spatial dimension of systemic criticality by determining the worthiness of protection of specific network elements. This judgement should ideally consider the result of a sectoral criticality assessment, which could, in principle, be performed on various spatial levels depending on the extent of the respective infrastructure system. Without an existing legal basis, a discourse-based approach among relevant stakeholders could evaluate the risks which were identified by a factual critically assessment. The authors have initiated such community-based approaches in Tanzania and Peru as further application tests of the presented methodological concept.

Current management approaches broadly neglect the criticality perspective. So does the Floods Directive (Council Directive 2007/60/EC), which only considers the concept of place-based vulnerability (so-called adverse consequences, see Art. 4 § 2 d). In February 2020, the BMI officially initiated the formal plan approval procedure of the BRPH. This plan is to establish a flood protection concept from a national and European perspective considering systemic criticality in Germany for the first time.

The introduction of a criticality-based approach in spatial planning represents the departure point for a process of rethinking spatial development at all levels and in all affected coastal and inland areas. The added value results from the new duty to consider both, the vulnerability and systemic criticality of protected objects, in future planning. Thus, there is a conceptual and semantic convergence between spatial planning and flood risk management planning under the Floods Directive.

## Conclusion

Understanding systemic criticality adds to the comprehension of impacts and response options of extreme events and climate change. Mega-disasters indicate that the larger share of economic losses from disastrous events is caused outside exposed areas, which are the focus of disaster risk management and climate change adaptation. Increasingly complex interdependences within the CI sos are the reason for this. Knowledge about systemic criticality of a subsector informs the understanding of the far-reaching consequences the impairment of a system component may have. Being able to make assertions towards the potential cascading effects from a disruption of critical services supports evaluating the risks associated with weather extremes better. With CI being highly vulnerable towards these extremes and them becoming more frequent and intense as the climate changes, understanding, assessing, and evaluating the systemic criticality of CI are a prerequisite for society to adequately cope with disastrous events by implementing effective risk management and adaptation policies.

The CI sos is not static but is continually evolving. The question as to how cascading effects change in the future remains. Some uncertainties might even be related to climate mitigation. For example, as society is moving away from combustion engines towards electric mobility, the electricity subsector gains even more centrality within the weave of systemic dependencies, while the petroleum subsector loses relevance. Since society is abandoning fossil fuels for renewable energy at the same time, this simultaneously increases the dependence of the entire transport sector on a functioning renewable energy sector, which in turn must expand significantly (International Energy Agency 2020) to meet the demand.

While there are uncertainties regarding the future development of the CI sos, including systemic criticality in risk evaluation can also reduce uncertainties in times of evolving hazards due to climate change. It can support anticipating probable worst-case scenarios of CI failure because it improves the understanding of the system-of-systems’ behaviour towards shocks. This evidence may support implementing measures to better cope with service disruptions, which society ultimately cares more about than the facility itself. Among these measures should be an increase in system redundancies providing the most critical services, e.g. electricity supply and ICT. Additionally, understanding the interdependencies between subsectors is crucial to engage all relevant stakeholders to improve communication and eventually break down silos in addressing disaster response and prevention.

The current Covid-19 pandemic and its expected losses from lost economic output show that international supply chains and dependencies of services spanning countries and continents may exacerbate financial damages when a hazard is not locally confined but ubiquitous. The more globalised supply chains become, the higher is the complexity of dependencies. A response to better prepare for future pandemics is to reduce global dependencies. Increasing redundancies, unravelling supply chains, and focusing on regional production and distribution processes can reduce global dependencies. In addition to working with local suppliers, such a development may be able to reduce the economic impact of ubiquitous hazards and would be—as a side effect—a valuable contribution to CO2 mitigation.

The key risks included in the most recent IPCC synthesis report include “[s]ystemic risks due to extreme weather events leading to breakdown of infrastructure networks and critical services” (IPCC 2014), dealing with which is highly relevant. This paper discussed novel approaches to assess system-internal effects on the CI system impacted by external climate change-driven hazards. These approaches are independent of the place-based concepts of vulnerability and risk by focusing on the functional character and inter-dependencies of the sos. Still, systemic criticality is a relatively recent research topic. There is much potential to continue research on its conceptualisation and operationalisation, which is necessary to address risks to CI more comprehensively eventually.

## Data Availability

Not applicable.
